# Purinergic Signaling at the Crossroads of Inflammation, Microbiota, and Intestinal Fibrosis

**DOI:** 10.1016/j.jcmgh.2026.101725

**Published:** 2026-01-16

**Authors:** Jiawen Shou, Ting Fu

**Affiliations:** Pharmaceutical Sciences Division, School of Pharmacy, University of Wisconsin–Madison, Madison, Wisconsin; Pharmaceutical Sciences Division, School of Pharmacy, University of Wisconsin–Madison, Madison, Wisconsin; University of Wisconsin Carbone Cancer Center (UWCCC), Madison, Wisconsin

Intestinal fibrosis is among the most intractable complications of inflammatory bowel disease, particularly Crohn’s disease, in which progressive extracellular matrix deposition leads to luminal narrowing, tissue stiffening, and ultimately the need for surgical intervention.^1^^,^^2^ Despite remarkable advances in anti-inflammatory and biologic therapies, no approved treatment effectively prevents or reverses established intestinal fibrosis, underscoring a critical unmet clinical need. A central challenge in the field remains understanding how chronic inflammatory signals are translated into sustained mesenchymal activation and fibrotic remodeling. In a previous issue of *Cellular and Molecular Gastroenterology and Hepatology*, Beatriz Elias Ribeiro and colleagues^3^ identify the purinergic receptor P2X7 as a key molecular mediator linking tissue injury, microbial dysbiosis, inflammasome activation, and intestinal fibrogenesis. Their findings position purinergic signaling not merely a bystander in chronic inflammation, but as an active driver of fibrotic remodeling in the intestine, providing a conceptual framework that connects extracellular adenosine triphosphate (ATP) sensing to long-term structural disease progression.

Extracellular ATP is a prototypical damage-associated molecular pattern released during epithelial injury and cellular stress.^4^ P2X7 receptor, a low-affinity ATP-gated ion channel, is preferentially activated under these conditions and has been extensively studied for its role in innate immune activation and inflammasome signaling.^5^ However, its contribution to intestinal fibrosis has remained incompletely defined. By integrating analyses of human intestinal tissues with complementary in vitro mechanistic studies and in vivo models of chronic colitis, the authors address this gap and identify P2X7 as a direct regulator of fibroblast behavior.

A major strength of the study lies in its translational relevance. Colonic biopsies from patients with Crohn’s disease reveal increased colocalization of P2X7 with α-smooth muscle actin-positive myofibroblasts, directly implicating purinergic signaling within fibrogenic niches of the diseased intestine. Complementary in vitro experiments demonstrate that ATP-P2X7 activation in human fibroblasts induces calcium influx, enhances migratory capacity, and increases collagen production, all hallmarks of myofibroblast activation. These findings support a model in which extracellular ATP functions not only as an immunologic danger signal, but also as a direct driver of mesenchymal remodeling.

The in vivo relevance of these observations is convincingly demonstrated using a chronic dextran sodium sulfate colitis model. Genetic deletion or pharmacologic inhibition of P2X7 markedly attenuates intestinal wall thickening, tissue stiffness, and collagen accumulation. These structural improvements are accompanied by reduced activation of the NLRP3 inflammasome, decreased caspase-1 expression, and lower levels of proinflammatory and profibrotic cytokines, including interleukin-1β and transforming growth factor-β. Together, these data firmly place P2X7 upstream of inflammasome-driven fibrogenic signaling in chronic intestinal inflammation.

An additional and particularly intriguing aspect of the study is the link between P2X7 signaling and the gut microbiota. P2X7 deficiency is associated with distinct microbial profiles and partial preservation of microbial diversity during chronic colitis. Although the directionality of this relationship remains to be fully elucidated, the findings raise the possibility of a feed-forward circuit in which dysbiosis, epithelial injury, extracellular ATP release, and inflammasome activation reinforce one another to sustain fibrotic remodeling. This concept aligns with emerging evidence that microbial cues shape mesenchymal cell function and fibrotic outcomes in the intestine.

The study further highlights a reciprocal relationship between P2X7 activation and peroxisome proliferator-activated receptor gamma (PPAR*γ*), a transcriptional regulator with well-established anti-inflammatory and antifibrotic roles in the intestine.^6^ Reduced PPAR*γ* expression in P2X7-competent settings suggests that purinergic signaling may suppress endogenous protective pathways that normally restrain fibroblast activation. This observation opens a door to therapeutic strategies that combine inhibition of danger signaling with restoration of transcriptional programs that counter fibrosis.

Collectively, this work reframes P2X7 as a central molecular hub that integrating tissue damage, immune activation, microbial dysbiosis, and mesenchymal remodeling. From a translational perspective, the findings are particularly compelling, given the availability of small-molecule P2X7 antagonists and prior clinical experience targeting purinergic pathways in inflammatory disorders.^7^^,^^8^ Future studies will need to define the cell type- and tissue-specific roles of P2X7, clarify its impact on epithelial repair, and determine whether temporal targeting can uncouple antifibrotic efficacy from immune compromise. Nonetheless, this study provides a strong conceptual framework positioning purinergic signaling as a promising therapeutic entry point for intestinal fibrosis.
